# Morphotype Classification Criteria and Influence of Sociocultural Factors on Perceived Shea Tree (*Vitellaria paradoxa* C.F. Gaertn) Natural Variation across Parklands in Benin

**DOI:** 10.3390/plants11030299

**Published:** 2022-01-24

**Authors:** Michée Fustelle F. Merinosy, Enoch G. Achigan-Dako, Paul Césaire Gnanglè, Eugène Kassa, Jean-Marc Boffa

**Affiliations:** 1 Laboratory of Genetics, Biotechnology and Seed Science, Faculty of Agronomic Sciences, School of Plant Science, University of Abomey-Calavi, Abomey-Calavi BP 2549, Benin; fustellemerinosy@gmail.com (M.F.F.M.); eugenekassa@mail.com (E.K.); 2 Institut National des Recherches Agricoles du Bénin, Cotonou BP 884, Benin; gnanglepaulcesaire2016@gmail.com; 3 World Agroforestry Centre, Nairobi 00100, Kenya; J.M.Boffa@cgiar.org

**Keywords:** *Vitellaria paradoxa*, morphotypes, criteria, sociodemographic attributes, West Africa

## Abstract

Trait diversity is crucial in undertaking the domestication of useful species such as *Vitellaria paradoxa* which makes a significant contribution to the rural household economy in Africa. This study aims to document the criteria farmers use to distinguish shea trees; how they vary according to age, education level and sociolinguistic group; and their perception of trees’ abundance and production. We surveyed 405 respondents across shea parklands in Benin using a semi-structured questionnaire. We used the Kruskal-Wallis test to evaluate the influence of sociodemographic attributes on relative criteria citation frequency and principal components analysis to characterize farmers’ perception on morphotypes’ abundance, fruits, and butter yields. The five most cited criteria were fruit size (55.5%), tree fertility (15.40%), bark colour (10.51%), timing of production (5.38%), and pulp taste (3.42%). The citation frequency of criteria varied significantly depending on the sociodemographic factors considered. Trees having small fruit (‘Yanki’) were reported to be widespread and high fruit/nuts and butter producers. Farmers perceived five important traits with variable importance depending on the sociocultural factors studied. This finding is a key step toward the development of a shea improvement program that could focus on the morphotype Yanki reported to potentially be a high fruit and butter producer.

## 1. Introduction

Wild edible fruit trees are the primary alternative sources of income during periods of food deficit in rural communities [1,2] in Africa. Wild tree foods are essential components of many African diets, especially in periods of seasonal food shortage [3]. Moreover, such plants are valuable genetic resources for new crop development [4,5]. Unfortunately, some of them are vulnerable or threatened by factors such as overexploitation (for firewood and charcoal), habitat fragmentation, climate change (variability in fruit production), and invasive and parasitic species (*Agelanthus* spp. and *Tapinanthus* spp.). Long-term utilization of those resourceful trees requires an informed strategy for conservation. To this end, rural communities‘ knowledge of their environmental resources has proven vital not only for conservation but also for the domestication of wild tree species with high economic potential [6]. Indigenous knowledge of species’ traits can serve as a valuable starting point for understanding natural variations in key phenotypic traits [7]. Based on these traits, farmers recognize different morphotypes in a given species [8]. Moreover, farmers prioritize certain morphotypes of a given wild species over others. For example, trees with robust growth patterns and desirable fruit and nut traits are deliberately selected and protected by farmers [9]. It is subsequently expected that local people would have some local selection criteria for individual trees presenting interesting characteristics which might be particularly targeted for collection, not only for consumption and other uses but also for conservation and possibly cultivation [10]. Therefore, understanding local knowledge systems and preferences can inform the selection and domestication of useful tree species and the development of plant improvement programs to increase local incomes and food and nutrient availability. In tropical Africa, wild fruit tree species of high economic importance include *Vitellaria paradoxa* C.F. Gaertn (Sapotaceae), commonly known as the shea tree.

*Vitellaria paradoxa* is a prominent multipurpose species, stated to cover a 500–750 km wide area stretching 6000 km from Senegal/Guinea to South Sudan and Uganda in 21 countries [11]. It is the only Sapotaceae tree species living on dry soil in the Sudanian and Sudano-Guinean phytochoria [12]. Shea trees are allogamous. Flowers appear between 10–15 years. The tree attains full fruit production after 45–50 years. The pulp of the fruit, which is normally sweet, is an important source of nutrients for humans, other mammals, birds, and bats. Each fruit generally carries one or rarely two nuts [13]. The shea tree is generally stocky, 10 to 15 m high, with a short bole (3–4 m) which can reach rather large diameters (80 cm and more). It is easily recognizable by its gray or blackish bark with thick and square scales. Unlike young plants, older plants have a woody bark that is deeply cracked into more-or-less rectangular plates reminiscent of crocodile’s skin [14]. The edge of the bark is reddish and exudes latex that is found in all parts of the plant. The fruit is an ovoid, 4–5 cm long, yellowish-green drupe. The nut contains an almond whose fat content (referred to as shea butter) is close to 50%. The tree is mostly valued for its oil-rich nuts [15,16,17]. Shea is a widely used traditional staple cooking oil. The average consumption of its oil is estimated at 21 g per person per day in rural areas [18]. In Benin, shea ranks among the top local wild edible trees that farmers protect or grow in their fields, and is mostly used for food processing and ceremonies [19]. Shea tree is also exported for use as a primary cocoa butter equivalent in the chocolate industry and a high-quality ingredient in cosmetics. The European Union’s authorization to use up to 5% shea in chocolate products has resulted in a significant growth in international demand in the last twenty years [18]. The local sale of fresh shea fruits, kernels, and butter also provides an alternative source of income to households in rural communities, particularly for women, who are the main collectors and processors of shea nuts, and retailers of shea products. In Benin, shea products contribute up to 36 to 46% of the income of rural households in the Atacora, Donga, and Borgou regions [20]. Apart from its use for cooking, shea oil is also used in traditional medicine (nasal decongestant, treatment of wounds, and child birth facilitation), in cosmetics (soap making, skin and hair moisturizers, and skin lotion) and for rituals, such as in traditional marriages as reported by Gwali et al. [21] in Uganda. Shea trees provide shade for farmers, herders, and their animals, and the ripe mesocarp (fruit pulp) is a key source of energy during the preparation of fields in the rainy season when grain supplies from the previous season are low [15,22,23]. The roots and bark also have numerous medicinal uses in the treatment of dysentery, suppurating wounds, and other ailments [22]. Due to its importance, shea has been protected through participatory management involving a complex mix of traditions and customs [21].

Despite the importance of this species for thousands of households in Benin, shea densities are declining, and shea populations are ageing. This state of things is due to extended forest land clearing and the discontinuation of the fallowing spell required for the natural regeneration of shea. Other causes include agricultural land expansion and the mechanization for commercial crops, tree cutting and removal out of crop fields, and firewood and charcoal production [9,23]. Moreover, shea resource degradation results in a loss of agricultural diversity and implicitly a potential loss of currency in the sector [24].

Studying the species’ diversity and documenting traditional knowledge and local classification systems are prerequisite to support conservation action and crop improvement [13]. They are instrumental in understanding phenotypic variation and help guide the selection of ethnovarieties that address local priorities. Theories on age, gender, and dynamics of knowledge hypothesis suggest that people’s socio-cultural and demographic attributes, such as gender, age, and ethnicity, influence their preference of a given species [25]. Moreover, people’s preferences for a given species determine their conservation attitudes toward that species [26]. Tietiambou et al. [6] also reported that attitudes toward the conservation of species vary according to the socio-demographic profiles (gender, age, education, ethnicity) of local people, their geographical location, and preferences based on use and market values. 

In Benin, ethnobotanical studies were conducted on some wild fruit tree species such as tamarind (*Tamarindus indica* L.) [2], African locust bean tree [*Parkia biglobosa* (Jacq.) R.Br. ex G.Don] [27], baobab *(Adansonia digitata* L.) [10], and marula or cider tree [*Sclerocarya birrea* A.Rich.)] [28]. So far, very little has been done to document phenotypic diversity and local knowledge systems on the shea tree. The study by Glèlè et al. [29] analysed the structure of shea butter trees in parklands located in different climatic regions of Benin and found that shea butter trees in the Guinean zone have zone developed large crowns but produce low quantities of fruits, whereas in the Sudanian regions, the opposite trend was observed. Gnanglè [30] reported that shea is an economically, socially, and culturally important tree for northern and central Benin. In addition, Agundez et al. [31] have recently studied local preferences for shea nut and butter production in Northern Benin. These authors found that the willingness to pay for a specific quality of nuts/butter depends on a number of their physical attributes. On local markets in Benin, the color, length, and weight of the nuts, as well as the color, smell, and texture of shea butter significantly influence, respectively, the processors’ willingness to accept and the consumers’ willingness to pay for a specific quality level. To date, no study has been undertaken to assess local knowledge on shea morphotypes traits and how they can be used in shea cultivars’ conservation and improvement.

This study documented folk knowledge of shea tree morphotypes, including classification criteria, nomenclature, farmers’ perceptions relative to shea morphotypes’ abundance and yields in Benin; it also unraveled the underlying sociodemographic factors that influence folk knowledge across the shea tree range in Benin. We hypothesized that (i) shea classification criteria by farmers are based on both fruit and tree traits, and (ii) shea morphotype knowledge is influenced by socio-demographic factors such as age, gender, instruction level, and sociolinguistic groups in Benin. 

## 2. Results

### 2.1. Identification and Characterization of Shea Morphotypes

#### 2.1.1. Criteria Used by Farmers for Shea Morphotypes Identification and Classification

Overall, farmers identified 35 different traits based on 16 primary criteria related to fruit, tree, and nut variants (Table 1). They mentioned 11 traits based on fruit characteristics (size, shape, fruiting period, and yield). Five traits were identified primarily according to characteristics of the fruit pulp (e.g., taste and density). Ten traits were identified based on tree characteristics (bark colour, height, scales size, heartwood colour, and trunk shape) and another five were identified based on shea nut traits (size, presence, and number of seeds). Farmers also differentiated shea trees based on leaf colour, which could be light or dark green. The top five classification criteria reported by farmers were fruit size (56.47%), tree fertility (15.67%), bark colour (10.70%), fruiting period (6.34%), and pulp taste (3.48%). Farmers reported 13 shea morphotype traits based on the top five criteria across the shea parklands zones in Benin. Some criteria were seldom used. Traits with a citation frequency lower than 1% were not further considered for the analysis.

Names for the shea tree in the sociolinguistic group of surveyed areas include Sombou (Bariba), Boulanga (Dendi), Tam (Yom), Wougo (Fon), Egui èmè (Tchabè), Kouli (Boko), and Mouta Koacha Tamou (Ditamari). Table 2 presents the local names of the most-mentioned traits by farmers in the different zones. These names, recorded across the shea parks of Benin, were based on morphological and organoleptic traits. Shea tree traits according to local communities were mainly related to bark colour, fruiting period, and fruit size; other traits were based on tree fertility and pulp taste. Farmers distinguished between shea trees that have white, black or red bark; they also differentiated between early fruiting trees (they bear fruits before the beginning of the rainy season), trees that have normal fruiting times (they produce fruits during the rainy season) and late-fruiting trees (they fructify after the two previous ones). Some trees produce fruits with sweet or insipid pulp, and others produce large, small, or medium-sized fruits. Farmers also reported that there are male and female shea trees. Local people distinguished between the two types based on the fruiting ability of shea tree: the “female”, fruit-producing trees, and the “male” trees, which never produce fruits. However, in the Ditamari sociolinguistic group, early maturing morphotypes were referred to as “Nda”, which means “male”; this comparison was borrowed, according to them, from the rule of spermatozoa during fecundation. In the Ditamari community, the production of fruit in shea trees is comparable to the conception of a child; the male seed (spermatozoon) gives fruit very early to form the embryo, whereas the female keeps pregnancy (the embryo) for several months before giving birth. That is why they equate the first to male and the second to female.

#### 2.1.2. Diversity of Shea Morphotype Traits in the Shea Parklands of Benin

Local knowledge on shea morphotypes traits diversity and Shannon index diversity varied from one zone to another (Table 3). Based on the classification criteria used, different numbers of morphotype traits were recorded across parks. Our results indicated that traits richness was greater in the Bembèrèkè, Parakou, Kandi, and Savè zones than in the Bohicon shea parkland zone.

**Figure 1:**
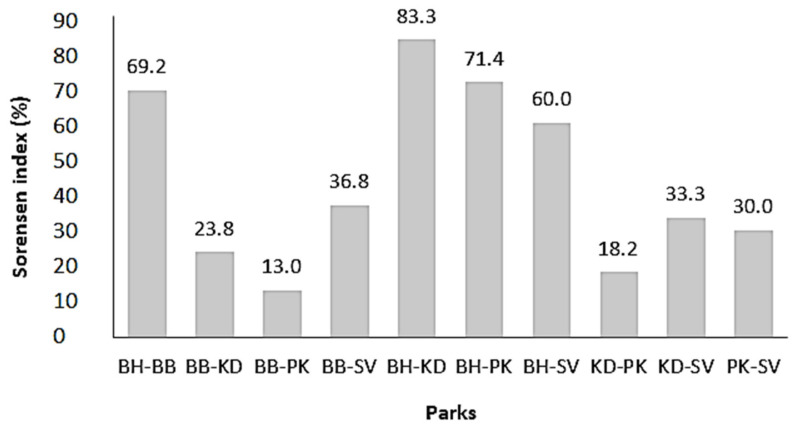
Bar plots showing the pairwise dissimilarity index between the five shea parks. BB— Bembèrèkè; BH—Bohicon; KD—Kandi; PK—Parakou; SV—Savè.

The pairwise analysis allows for the identification of patterns of dissimilarity between zones based on Sorensen index. Sorensen index values range from 13.0 to 88.3% (Figure 1), taking into account ten combinations of pairwise comparison: Bembèrèkè–Bohicon (BB-BH), Bembèrèkè–Kandi (BB-KD), Bembèrèkè–Parakou (BB-PK), Bembèrèkè–Savè (BB-SV), Bohicon–Kandi (BH-KD), Bohicon–Parakou (BH-PK), Bohicon–Savè (BH-SV), Kandi—Parakou (KD-PK), Kandi–Savè (KD-SV), and Parakou–Savè (PK-SV). The dissimilarity in terms of shea traits’ natural variation between northern zones (Parakou, Bembèrèkè, and Kandi) is rather low (<25%). In contrast, dissimilarity index values were higher between northern and southern (Bohicon) zones (60–88.3%). There is a big difference in local knowledge between the northern zones and southern zone. 

**Figure 2:**
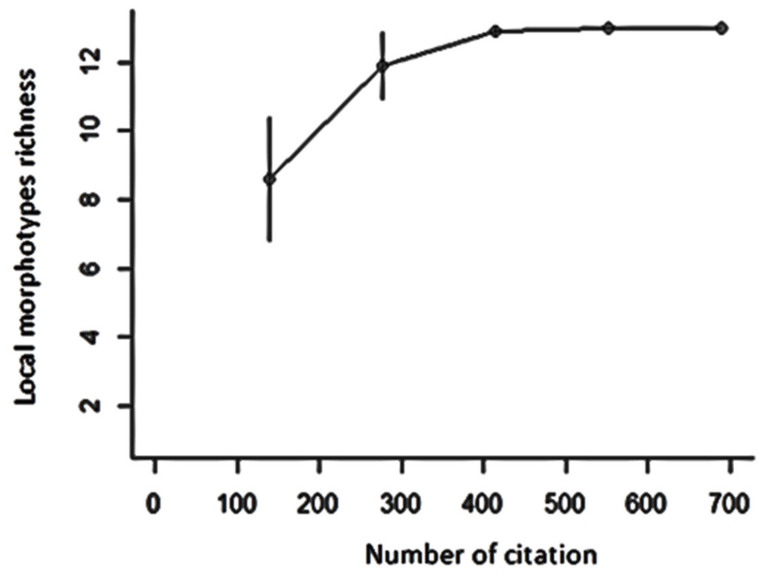
Cumulative curve of morphotype richness in shea based on number of citations.

In order to understand how the richness of the morphotype traits varied according to the number of zones considered, we realized the cumulative curve of morphotype richness (Figure 2). The curve increases in proportion to the areas explored up to an asymptotic threshold, which means that the shea morphotype richness increases as more areas are surveyed and becomes invariable when the entire range has been considered.

#### 2.1.3. Characterization of Shea Morphotypes’ Trait Performance

**Figure 3:**
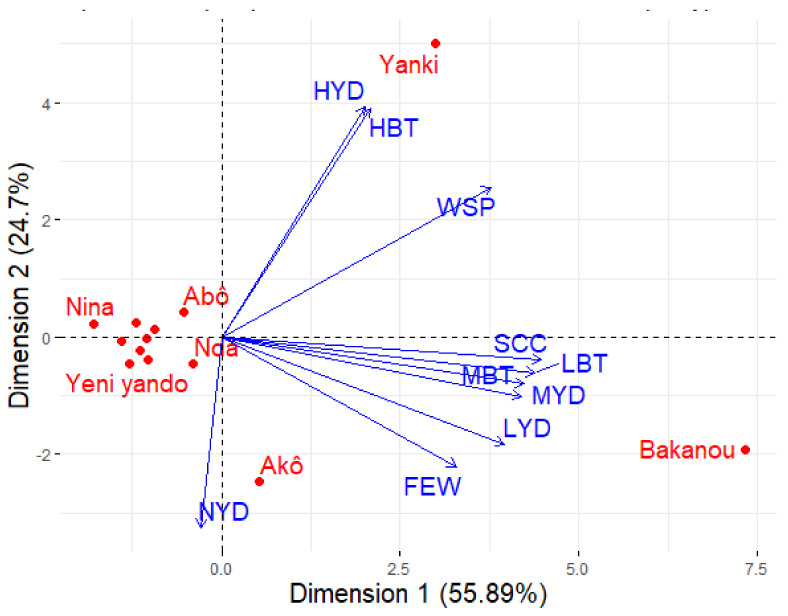
PCA biplot showing shea morphotypes and their frequency of distribution, fruit, and butter yield characteristics. Yanki = Fruit with small size; Bakanou = Fruit with large size; Saganin = Fruit with medium size; Akô = Male shea tree; Abô = Female shea tree; Kpika = White bark; Wonka= Dark bark; Soarou = Red bark; Nda = Precocious production; Nina = Normal production; Niendembou = Late production; Dourobou = Sweet; Yeni yando = Insipid. HYD = High fruit production; MYD= Average shea fruit production; LYD = Low shea fruit production; NYD = No fruit production; HBT= High amount of butter; MBT = Average amount of butter; LBT = Low amount of butter.

Respondents reported three different levels of shea morphotypes’ abundance attributes, such as ‘widespread’, ‘few’, and ‘scarce’. Trees’ fruit yield and butter yield after processing could be, according to respondents, ‘high’, ‘medium’, or ‘low’. Principal component analysis (PCA) performed on the perception scores of the distribution, morphological, and performance traits showed that the first two dimensions accounted for 80.59% of the observed variation (Figure 3). The first component is positively correlated with the variables of medium and low yield (fruit yield), medium and low quantity of butter after processing (medium butter and low butter), and scarce and few distribution frequencies. The second component is negatively correlated with the variable null yield (no fruit production), and positively correlated with the variables high yield, high butter, and widespread.

According to the biplot of morphotypes and abundance, fruit yield, and butter yield characteristics (Figure 3), it appears that, based on farmers’ perceptions, shea morphotypes having the trait Yanki (small fruit) are characterized by high fruit yield, a high amount of butter after nut processing, and are widespread. On the other hand, shea morphotypes with the trait Bakanou (large fruit) are rare (scarce) or very little spread (few). They may have medium or low fruit production, and the nuts inside their fruits produce an average amount of butter after processing. The morphotypes characterized by the trait Akô (male tree) do not have the ability to produce fruit (null yield). The other morphotype traits do not reveal any particular characteristics. No specific trend of these variables has been recorded for the other morphotype traits.

### 2.2. Influence of Occurrence and Sociocultural Factors on Local Knowledge of Shea Morphotypes’ Natural Variation

The average number of traits cited by each respondent did not vary significantly (*p* > 0.05) regarding the parkland, age, gender, instruction level, or sociolinguistic group. 

**Figure 4:**
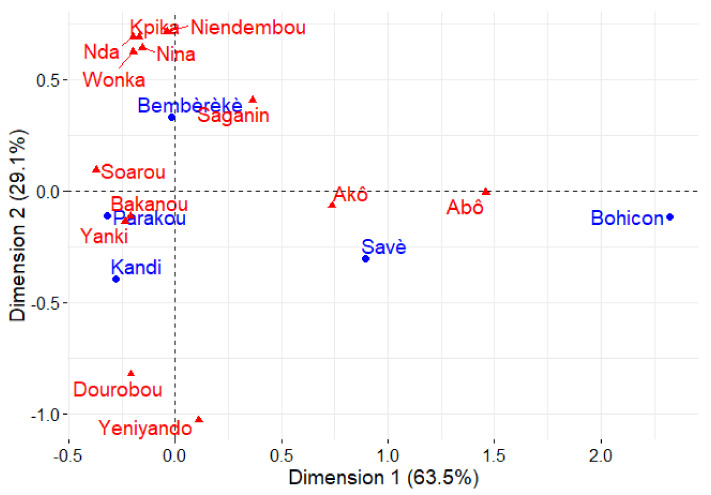
Factorial correspondence analysis biplot showing the main traits cited in the five shea parklands zones.

However, the relative frequency of citation of the 13 main local morphotypes varied very significantly regarding the shea parkland zones (*p* < 0.001). The factorial correspondence analyses (FCA) plot (Figure 4) shows the traits most mentioned by farmers in each zone. Morphotypes that have red bark (Soarou), and large or small fruit size (Bakanou and Yanki) were more common in the Parakou and Kandi zones. Local morphotypes identified based on the pulp taste, such as sweet and insipid (Dourobou and Yeniyando), were more often mentioned in Kandi park than in others. Morphotype traits relative to the timing of production, medium fruit size (Saganin), and bark colour (specifically morphotypes having white and black bark) were common in the Bembèrèkè shea parkland zone. On the other hand, the morphotype traits Akô and Abô were mostly cited in the Savè and Bohicon zones.

**Figure 5:**
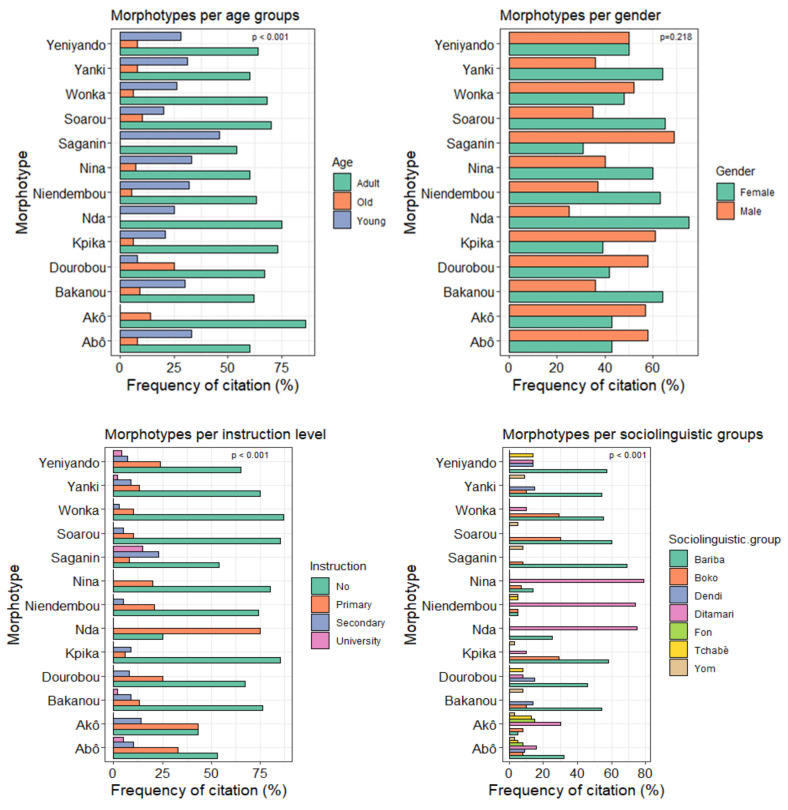
Relative citation frequency of the 13 main local traits according to age, gender, instruction level, and sociolinguistic group.

With exception to gender (*p* = 0.218), all socio-demographic characteristics very significantly (*p* < 0.001) influenced the relative frequency of the 13 main traits of shea morphotypes (Figure 5). Figure 5 shows the relative citation frequency (RCF) of traits according to age group, gender, instruction level, and sociolinguistic group. All traits were known to the three age groups; however, local knowledge varied from one age group to another. The relative frequency of citation decreased from adults to old people, with the count being surpassed by the young for all traits except the trait “Dourobou” which was more often reported by elders than young respondents. 

There was a trend of the influence of instruction level on the decrease of local knowledge on morphotypes traits from illiterate to literate respondents. Morphotype citation frequency was higher for illiterate (25 < RCF < 85%) respondents and respondents with primary school level education (1 < RCF < 80%) than those of respondents who attained secondary school or university education (0 < RCF < 25%). Moreover, traits relative to bark color were unknown to respondents that reached university.

The relative citation frequency of traits also varied from one sociolinguistic group to another. All sociolinguistic groups cited the female trait Abô. Except Dendi people, all sociolinguistic groups recognised the trait Akô, which is qualified as male by farmers. The Bariba sociolinguistic group cited all traits, but mainly those related to fruit size (Yanki, Saganin and Bakanou), bark colour (Kpika and Wonka), and pulp taste (Dourobou and Yeniyando) with RCF > 50%. The Fon sociolinguistic group mentioned only female and male traits. Shea morphotype traits identified based on the timing of the fruit production (Nina, Nda, and Niendembou) were registered mostly within the Ditamari sociolinguistic group (70 < RCF < 80%). The differentiation of morphotypes according to fruit size, pulp taste, and bark colour criteria were more common to the Bariba, Dendi, Yom and Boko sociolinguistic groups located in the northern part of the country.

## 3. Discussion

### 3.1. Classification Criteria of Shea Morphotypes in Benin

Farmers reported several classification criteria across shea parkland zones based on their importance. Fruit size, tree fertility (male and female), bark colour, timing of production, and pulp taste were the top five most reported classification criteria. Among those criteria, fruit size was the first one mainly used to distinguish morphotypes. This result confirms our hypothesis that shea tree classification is mainly based on fruit variants and is consistent with the findings of Gwali et al. [32] in Uganda and Karambiri et al. [13] and Sandwidi et al. [14] in Burkina Faso. Ekué et al. [33] also reported for *Blighia sapida* K.D. Koenig that differentiation criteria included fruit size, which was by far the most quoted criterion by farmers in Benin. Similarly, in a study on farmer classifications of the baobab tree (*Adansonia digitata*) in West Africa, Assogbadjo et al. [10] recognized ‘male’ and ‘female’ baobab trees. These authors also reported that local perceptions of baobab differentiation vary from one country to another. For instance, in Uganda and Burkina Faso, local shea classification was not based only on fruits, but also on nut variants. This finding revealed that variation can be observed in classification criteria depending on the geographical or sociolinguistic group. Indeed, local communities from the Bohicon and Savè zones with Fon and Tchabè as their dominant sociolinguistic groups have commonly distinguished male and female morphotypes (Figure 4). Moreover, the differentiation of morphotypes according to the bark colour criterion, among others, was more common to the Bariba, Boko, Ditamari, and Yom sociolinguistic groups which belong to the Bembèrèkè zone. As the result of the above, it seems that, contrary to what Sandwidi et al. [14] have observed in Burkina Faso, certain sociolinguistic groups in specific area in Benin were able to identify individual shea trees by merely looking at the bark colour of the trees. This result confirms the first hypothesis of this study concerning the use of tree characteristics to classify morphotypes. Assogbadjo et al. [34] reported that farmers can also distinguish different types of baobab trees using their own criteria based on the colour of the bark, among other things. Furthermore, utilization of fruit variations as the main description criterion could be linked to the fact that the description of the tree is related to the most important tree features for producers. Indeed, in the Bohicon zone, farmers distinguished trees based on fertility, which is linked to the size and vigor of the tree. They also talked about the heartwood of the tree and the ease of cutting. In Bohicon parkland, farmers mainly use the tree for charcoal (of higher quality according to them) and firewood, which leads to a loss in shea tree diversity. Farmers of that parkland reported that shea nut processing into butter is not practiced in their community. While in other sociolinguistic groups, the male morphotype is linked either to the capacity to produce the fruit, or to the seasonality of production. In those communities, the fruit is the most important part, even if felling shea trees for charcoal and firewood is moderate but not absent. 

The usefulness of collecting local communities’ knowledge relies on the fact that such information could help determine the true value of the species, leading to more rational decisions about its sustainable utilization [27]. Based on our findings, conservation actions have to focus on each sociolinguistic group’s preferences. In addition, future prospects have to investigate traits preferred by farmers and end-users along shea value chains. It is also imperative to characterize shea morphotypes identified by respondents across their distribution range in Benin in order to confirm local perceptions and evaluate traits’ plasticity or stability for selection of mother trees to be used in domestication programs. Moreover, due to the demographic pressure and reduction of fallows, there is a need to safeguard shea trees’ natural variation through sensitization of farmers to the sustainable management of shea resources. The promotion of assisted natural regeneration in protected areas across its distribution range can be an endeavor of the national forest management and reforestation projects.

### 3.2. Diversity of Shea Classification Traits Cited by Farmers

Overall, farmers identified 35 different shea morphotypes based on 16 primary criteria relative to fruit, tree and nut variants. The number of morphotypes reported in literature varies from one country to another and from one area to another inside the same country. According to Karambiri et al. [13] who obtained a lower number of ethnovarieties in comparison with the Gwali et al. [32] study, the high number of ethnovarieties identified in a study could be linked to the size of the study area. This means that the bigger the study area, the higher the number of morphotypes traits identified. Morphotypes’ richness increases according to the number of citation and zones at first and then becomes stable when the maximum number was reached for the five zones (Figure 2). In other words, to assess the whole diversity, it is necessary to go through all of the shea distribution range. Indeed, Akohoué et al. [35], who recorded five Kersting’s groundnut (*Macrotyloma geocarpum* (Harms) Maréchal & Baudet) landraces in Benin and Togo while previous authors reported three traditional cultivars only, explained that this is due to the fact that previous studies did not include all production areas, and as result, a part of the existing genetic diversity was left out. More recently, Coulibaly et al. [36] collected six different phenotypic groups of *Macrotyloma geocarpum* in Burkina Faso and Ghana. It appears, based on the analysis, that, till the whole distribution range of a species has been explored, the entire cultivar or morphotype diversity could not be documented. For this study, the diversity was greater in the Bembèrèkè zone and very low in the Bohicon (Table 3). This finding could be, among others, linked to the scale of the zone and tree density. Bembèrèkè shea parkland zone is the largest with the highest average density per hectare (43 trees/ha) [29,30]. To catch the whole diversity of shea morphotypes’ traits that hold importance for farmers, investigations could be extended to more villages inside shea parklands and different categories of actors. This will be useful in selecting traits to be prioritized for genetic studies.

### 3.3. Characterization of Shea Morphotypes

Knowledge of the agromorphological diversity of the identified shea trees is essential to begin any program of improvement of the species [37]. Since the most treasured product of the shea tree is the butter extracted from its kernels [38] which also depends on trees production, there is a need to assess the available diversity in terms of abundance of morphotypes and their fruit production yield and associated butter yield. This study revealed that farmers’ perception related to the abundance and the fruit and butter production of the Yanki morphotype is unanimous while for the Bakanou morphotype it varies. On the one hand, morphotype Yanki (with small fruits) was reported to be widespread and characterized by high yield and high amount of butter. Moreover, morphotype Bakanou (with large fruits) was reported to give an average amount of butter and was reported to be rare or very little spread. To date, no prior study on shea morphotype abundance had been conducted. For Fandohan et al. [39], rural communities’ knowledge on Tamarindus’ ecological range was congruent with scientific findings. This is why there is a crucial need to fill the gap for the shea tree to better understand the factors that influence this state of affairs which should meet farmers’ preferences. In addition, perceptions were scattered among farmers about this morphotype’s fruit production, which can be high, medium, and, sometimes, very low. Across the study areas, farmers associated fruit size with soil fertility, and as such, the rarity of these morphotypes could be linked to the low level of soil fertility in the area. A major problem for cropping systems in the tropics is the reduction in soil productivity that accompanies most systems of continuous cultivation [40]. The past decades have shown a rapid decline in land productivity and soil fertility in particular [10]. Moreover, the observed inter-annual variability in fruit yield [41] can explain the absence of consistency among farmers about these variables. In fact, the main constraints encountered with nut production are the remarkable decrease in production and its huge fluctuation from year to year [42]. Authors have hypothesized many combined biotic and abiotic factors underlying the annual variation of shea trees’ fruit production, but this process is not fully understood for now. Some questions raised from this study include: (1) are farmers’ perceptions on morphotypes’ abundance, fruit, and butter yields congruent with scientific knowledge? (2) What is the significance of the environmental component in the observed phenotypes?

### 3.4. Influence of Ecology and Sociolinguistic Attributes on the Knowledge of Shea Morphotypes Knowledge

Analysis of the determinants of shea morphotype knowledge shows that none of the socio-demographic characteristics significantly influence the number of shea morphotypes known to the respondent (Prob > 0.05). This result is in agreement with Karambiri et al. [13] who also reported a similarity across the villages they surveyed. There is therefore a minimum threshold of shea morphotype knowledge reached by almost all respondents regardless of their origin, age category, sociolinguistic group, and level of education. However, sociolinguistic groups strongly influenced the diversity of morphotype traits reported. For instance, the criterion timing of production was specifically more reported by Ditamari sociolinguistic groups than others and could be linked to the degree of interest carried by each specific group or area. For instance, Gwali et al. [32] have also found a significant influence of ethnicity on ethno-variety nomenclature and claimed that the variation in folk knowledge among the various ethnic groups may be due to the intensity of utilization of the shea tree and its products. In reality, each community faces different edaphic, climatic, and social bottlenecks and adapts their lifestyle and needs accordingly. In consequence, the need to adapt tree management practices is vital for peoples’ wellbeing [9]. 

Morphotype knowledge increased following the south and north gradient, and this is reflected by morphotypes’ diversity and specific richness (number of different kinds of morphotype traits) mentioned by zone. Based on these results, we can assert that shea morphotype knowledge was low in the Bohicon zone, medium in Savè, and high in the Parakou, Bembèrèkè, and Kandi zones. In addition, certain morphotype traits were specific to a given sociolinguistic group, as found by Fandohan et al., [43] for *Synsepalum dulcificum* (Schumach. & Thonn.) Daniell, which belongs to the same family as *Vitellaria paradoxa*. The intercultural differences observed in the knowledge of the uses of *S. dulcificum* are related to the environment and the availability of the plant resource [28]. Surprisingly, gender does not influence the frequency of citation of the most reported traits. Meaning that both men and women have similar knowledge of the shea morphotypes. However, recently, studies reported differences in men and women’s knowledge on plants. For instance, according to Laleye et al. [44] in a case study related to knowledge of plants in traditional treatment of diabetes in the Benin Republic, the fact that men cited more species than women may be due to the close association of men’s knowledge with the treatment of diabetes. In our study, the same level of knowledge of morphotypes between men and women reveals that shea butter is certainly of different but important interest for both men and women. Indeed, the level of knowledge of the species by women is justified, as they are the main actors from the beginning to the end of the entire shea production chain. Women control shea production as 92% of the collection and 98% of the processing activities were performed solely by women and girls even if shea fruits are consumed by both men and women [45]. Men also have the power to make decisions about the conservation of shea trees since they are the owners of lands that shelters shea trees. Gender influence could be perceived in the processing of shea nuts where men were not involved. Along the same lines, Agúndez et al. [31] suggested that the development of shea resource management and conservation programs should include ethnic preferences and consider gender, to avoid reducing women’s profits in the shea butter local market.

## 4. Conclusions

Our study provides insight into the most reported traits of *Vitellaria paradoxa* in the five shea parkland zones of Benin. The number of morphotypes cited by a respondent does not vary significantly according to sociodemographic factors, revealing that shea morphotype knowledge is almost the same across the shea parklands. However, classification criteria of morphotype were diverse and strongly influenced by age, instruction level, and sociolinguistic group. The peculiarities of certain zones and sociolinguistic groups about the classification criteria were underlined. This emphasizes the need to take into account sociocultural aspects to assess indigenous knowledge and, by implication, to identify indigenous preferences of shea morphotypes. The local classification of shea morphotypes is paramount for shea tree selection and improvement. 

As shea butter demand over the world is growing, a progressive transition from natural threatened stands to artificial plantations is crucial and has to be implemented. The results of this study are the first steps into that transition, as they provide a set of primary traits that are valuable for farmers. Future steps to operate the production transition include: (1) to expand the inventory of useful traits to the others stakeholders of the value chains and prioritize them with particular emphasis on sociodemographic attributes; (2) to evaluate the stability, and the genetic by environment effects on prioritized traits in order to select the most productive genotypes or morphotypes to serve as mother trees; (3) to reproduce elite morphotypes through vegetative reproduction methods that conserve the genetic material of the mother tree; and (4) to create shea botanical gardens and protected areas in national forests and parks to safeguard shea natural diversity for future generations. 

## 5. Materials and Methods

### 5.1. Study Area

**Figure 6:**
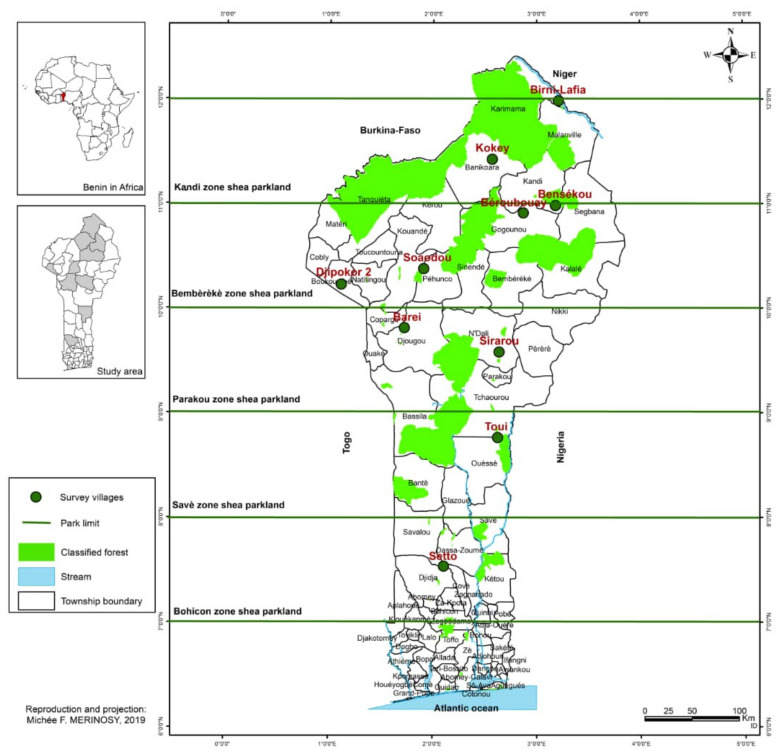
Surveyed areas across shea parks in Benin and major sociolinguistic groups (in brown italics).

The study villages are located in the shea parkland zones identified and described by Gnanglè [30] in Benin based on the North–South and East–West rainfall gradient, socio-cultural groups, plant production period, and soil types [30,46]. We chose villages in close collaboration with resource persons from the National Institute of Agricultural Research in Benin and shea industry (Fludor) staff. The choice of villages was proportional to the size and the dominant sociolinguistic groups of each of the shea parkland zones. In total, we surveyed ten villages in five shea parkland zones including Bohicon, Savè, Parakou, Bembèrèkè, and Kandi (Figure 6). 

The Bohicon zone shea parkland is located between latitudes 7° and 8° N and includes the entire shea population stretching from Bohicon to Dassa-Zoumè. The average annual rainfall in the park is 1200 mm. Three types of soil can be distinguished, namely the weakly desaturated ferralitic soils or ‘’terre de barre’’ that stretch south of the park, the impoverished tropical ferruginous soils encountered in the center of the park, and the hydromorphic soils north of the park. The average density of shea trees was 15 trees per hectare. The village of Setto was selected in this zone for the study. The dominant sociolinguistic group was Fon. 

The shea park of the Savè region is located between latitudes 8° and 9° N and covers the shea population stretching from Glazoué to south of Tchaourou. The average annual rainfall is between 1100 mm and 1200 mm. Soils are basically leached or depleted tropical ferruginous soils. The shea population of the Savè region has an average density of 26 trees per hectare. The village of Toui was selected in this zone for the study. The dominant sociolinguistic group was Nagot.

The shea parklands of the Parakou zone extend between latitudes 9° and 10° N and include all the shea population located between Tchaourou and N’dali and between Parakou and Djougou. It is an area characterized by a Sudanian climate with an average annual rainfall ranging from 1000 to 1200 mm. In some places, moderately desaturated ferralitic soils are found. The average density of the park was 26 trees per hectare. The villages of Barei, and Sirarou were selected in this zone for the study. The dominant sociolinguistic groups were Yoa and Bariba. 

The Bembèrèkè zone shea parklands are located between latitudes 10° and 11° N and includes all the shea population extending from Bembérékè to Gogounou. The average annual rainfall in this park is 1100 mm. The average density of the park was 25 trees per hectare. Four villages, namely Bensékou, Béroubouay, Soaodou, and Dipokor 2, were selected. The dominant sociolinguistic groups included Boko, Bariba, and Ditamari. Three types of soils can be distinguished in this park: tropical ferruginous soils slightly leached, whether concreted or not on kaolinic material. In the depressions, hydromorphic soils were found, while under a vegetative cover we found ferralitic soils moderately unsaturated.

The shea parklands of the Kandi zone are located beyond 11° N. It brings together the entire shea population ranging from Kandi to Malanville and from Kandi to Banikoara. This parkland receives an average rainfall of 800 mm. The average density of the park was 31 trees per hectare. The villages of Birni-Lafia and Kokey were selected. Dominant sociolinguistic groups in those villages included Dendi and Bariba. Soil types encountered included poorly evolved soils, tropical ferruginous soils slightly leached, and hydromorphic soils. 

The rural populations of the five zones host 1,637,434 inhabitants [47]. Livelihood activities carried out by the people of these sociolinguistic groups include agriculture, ranching, fishing, hunting, processing of agricultural products, trades, and crafts [43]. Women mainly practice the processing of agricultural products, individually or in groups, with rudimentary equipment. The main processed products are *Vitellaria paradoxa* nuts (processed into butter), seeds of *Parkia biglobosa* (processed into a food condiment), the grain of *Sorghum bicolor* (L.) Moench (processed into an alcoholic beverage and used in some traditional ceremonies), and groundnuts (*Arachis hypogaea* L.) processed into oil [43].

### 5.2. Socio-Demographic Profile of Respondents 

The characteristics of the study population varied according to the proportion of respondents’ ages, instruction levels, and sociolinguistic groups. Respondents ages varied from 19 to 90 years old. There were mostly adults surveyed in all parklands, and the majority of them were not educated. In the Bembèrèkè, Bohicon, and Savè zones, both genders were well represented, while in Kandi and Parakou, more women than men were surveyed (Table 4). 

In total, seven sociolinguistic groups were represented in the study. Six sociolinguistic groups were found in one municipality only. The Bariba sociolinguistic group was found in four survey villages (Sirarou, Soaodou, Beroubouay, and Kokey). The Fon and Tchabè/Nagot (in Setto and Toui villages) belong, respectively, to the subgroups “gbe” and “ede”, and both belong to the large linguistic group “Kwa”. The other five groups (Bariba, Boko, Ditamari, Yoa, and Dendi) belong to the large group of languages known as “Gur” or “Voltaïque” which comprise most of the languages of the northern part of the country [48].

### 5.3. Data Collection

The survey was carried out from January to March 2019. In the study area, we were granted verbal permission from traditional leaders before starting the surveys. Semi-structured interviews were conducted using the respondents’ preferred languages which were Fon, Nagot/Tchabè, Bariba, Boko, Yoa, Ditamari, or Dendi. To facilitate the communication between the respondents and the first author (where necessary), she was accompanied by a well-trained local guide (who understood both the interviewee-spoken language and French) in each village to facilitate the question/answer translation. About 39 to 45 farm household respondents were surveyed per village in ten villages, totaling 405 respondents. Respondents were chosen through a random walk in all hamlets of the village. However, when the selected participant was not available or did not want to be interviewed, the random walk continued until another consenting respondent was found. The first section of the questionnaire dealt with informants’ age, gender, sociolinguistic group, and education level. The second part focused on criteria used by the respondent to differentiate shea trees, the local names and their meaning, and the respondent’s perception on the morphotypes’ relative abundance, fruit yield, and butter yield after processing (Table S1). 

### 5.4. Data Analysis

We evaluated the relative frequency of sociodemographic attributes, especially age group, gender, sociolinguistic group, and level of education. We used a Fisher exact test to analyze contingency tables. We performed frequency distributions by using the number of citations of each criterion by all respondents and the total number of citations of all criteria to estimate proportion of main morphotype classification criteria cited by farmers and rank them from the most to the least important. Only variables exhibiting a relative frequency of citation of at least 1% (approximately 9 out of 405 respondents) were used in further analysis and interpretation. In addition, a detailed analysis of the diversity of shea morphotypes was carried out with a BiodiversityR package [49]. The variation in local knowledge on shea morphotype trait composition in zones was measured by computing a Sorensen index using betapart package in R. To characterize morphotype abundance and performance, we performed a principal component analysis (PCA) using respondents’ perceptions of morphotype traits. Variables included frequency distribution, fruit, and butter yields estimated using qualitative scales (e.g., low, medium, high).

To evaluate the influence of socio-demographic factors on local knowledge of shea morphotype traits, a generalized linear mixed model (with a Poisson error family) was performed using the number of morphotype traits known by the respondent as the response variable. Fixed factors included zones of occurrence, age group, gender, education level, and sociolinguistic group; village provenance was used as a random factor. We performed a Kruskal-Wallis test on the relative frequency of citation of morphotype traits per zone, age group, education level, gender, and sociolinguistic groups, with the package Agricolae. A factorial correspondence analysis was carried out to assess the most-cited morphotype traits across the parkland zones. All analyses and graphics were performed in an R 3.5.1 software environment (R Core Team, 2018).

## Figures and Tables

**Table 1:** Identification criteria of shea morphotypes used by farmers and associated traits.

Criteria	Traits	Number of Citation	Trait Relative Frequency (%)	Criteria Relative Frequency (%)
Fruit size	Small	218	27.11	
	Large	212	26.37	56.47
	Medium	24	2.99	
Tree fertility	Male	75	9.33	15.67
	Female	51	6.34	
Bark colour	White	34	4.23	
	Black	32	3.98	10.70
	Red	20	2.49	
Timing of production	Precocious	20	2.49	
	Normal	12	1.49	6.34
	Late	19	2.36	
Pulp taste	Sweet	15	1.87	
	Insipid	11	1.37	3.48
	Astringent	2	0.25	
Fruit shape	Oblong	7	0.87	1.24
	Ellipsoid	3	0.37	
Nut size	Small	7	0.87	1.62
	Large	6	0.75	
Fruit yield	Low	6	0.75	
	Medium	1	0.12	1.37
	High	4	0.50	
Tree height	High	5	0.62	1.24
	Low	5	0.62	
Nut presence in fruit	Absent	3	0.37	0.50
	Present	1	0.12	
Scales size	Big	2	0.25	0.24
Leaves colour	Light green	1	0.12	0.25
	Dark green	1	0.12	
Pulp density	Thick	1	0.12	0.25
	Thin	1	0.12	
Number of seeds	Twin	1	0.12	0.12
Heartwood colour	Brown	1	0.12	0.25
	White	1	0.12	
Trunk shape	Right	1	0.12	0.25
	Curled	1	0.12	

**Table 2:** Most-mentioned traits related to shea morphotypes in Benin.

N°	Local Names of Traits	Language	Main Criteria	English Meaning of Local Names
1	Kpika	Bariba	Bark colour	White bark
2	Wonka			Black bark
3	Soarou			Red bark
4	Nda	Ditamari	Timing of production	Precocious
5	Nina			Normal
6	Niendembou			Late
7	Abô	Tchabè (Nagot)	Fertility	Female
8	Akô			Male
9	Dourobou	Bariba	Pulp taste	Sweet pulp
10	Yeni yando			Insipid pulp
11	Bakanou	Bariba	Fruit size	Large fruit
12	Saganin			Medium fruit
13	Yanki			Small fruit

**Table 3:** Shannon index diversity and shea trait richness per park.

Park	Traits Richness	Shannon Index Value
Bembèrèkè	11	2.002
Bohicon	2	0.693
Kandi	10	1.394
Parakou	12	1.734
Savè	8	1.743

**Table 4:** Socio-demographic and sociolinguistic group characteristics of the survey population. n—number of respondents. Numbers in parentheses represent total number of respondents surveyed in each zone.

Variables	Modalities	Survey Zones									
		Bembèrèkè (161)		Bohicon (40)		Kandi (79)		Parakou (80)		Savè (40)	
Characteristics											
	Latitudes	9°/10°		7°/8°		>10°		9°/10°		8°/9°	
	Tree density/ha	25		15		31		26		26	
	Rainfall (mm)	1100		1200		800		1000–1200		1100–1200	
Socio-demographic factors											
		n	%	n	%	n	%	n	%	n	%
Age category	Young (< 30 y)	100	62	34	85	50	63	52	65	26	58
	Adult (30 ≤ A ≤ 60 y)	51	32	6	15	21	27	18	23	16	36
	Old (> 60 y)	10	6	0	0	8	10	10	13	3	7
Gender	Female	90	56	16	40	56	71	51	64	19	42
	Male	71	44	24	60	23	29	29	36	26	58
Instruction level	Illiterate	127	79	27	68	58	73	58	73	20	44
	Primary	17	11	8	20	18	23	18	22.8	8	18
	Secondary	14	9	5	13	3	4	3	4	12	27
	University	3	2	0	0	0	0	0	0	5	11
Sociolinguistic group											
	Fon	-	-	36	90	-	-	-	-	-	-
	Tchabè (Nagot)	-	-	-	-	-	-	-	-	12	27
	Bariba	80	50	-	-	36	46	33	41	-	-
	Yoa	-	-	-	-	-	-	35	44	-	-
	Boko	37	23	-	-	-	-	-	-	-	-
	Ditamari	40	25	-	-	-	-	-	-	-	-
	Dendi	-	-	-	-	33	42			-	-
	Others	4	3	4	10	10	13	12	15	33	73

## Data Availability

All data generated or analysed during this study are included in this published article (and its Supplementary Material).

## Supplementary Materials

The following are available online at https://www.mdpi.com/article/10.3390/plants11030299/s1, Table S1: Questionnaire.
