# Supplementary material for: Morphotype Classification Criteria and Influence of Sociocultural Factors on Perceived Shea Tree (Vitellaria paradoxa C.F. Gaertn) Natural Variation across Parklands in Benin
Source: Plants (Basel). 2022 Jan 24;11(3):299. doi: 10.3390/plants11030299 (PMC8838959; doi:10.3390/plants11030299)
Supplement: Supplementary file 1 [file plants-11-00299-s001.zip › plants-1407339-supplementary.pdf]

Table S1. Questionnaire

1. **Respondent information**

Name:………………………………………………...

Telephone number :…………………………….

Date:…………………………………..

2. **Sociodemographic characteristics of respondent**

Questions	Modalities	Response
1. Shea park	1=Kandi Park 2= Bembèrèkè Park<br>3=Parakou Park 4= Savè Park 5= Bohicon Park	
2. Department	1=Alibori 2=Borgou 3=Atacora 4=Donga 5= Collines 6=Zou	
3. Commune	1=Malanville 2= Banikoara 3=Kandi<br>4= Boukoumbé 5=Pehunco 6=Beroubouay<br>7=N'Dali 8=Djougou 9=Ouèssè 10=Djidja	
4. Village	1= Birni-Lafia 2= Kokey 3=Bensékou 4= Dipokor2<br>5=Soaodou 6=Berouboué ouest 7=Sirarou 8= Barei<br>9=Toui 10=Setto	
5. Age		
6. Sexe	0=Female 1= Male	
7. Niveau d'alphabétisation	0=Analphabète 1=Alphabétisé	
8. Instruction level	1= primary, 2= secondary, 3=university, 4= illiterate	
9. Sociolinguistic group	1=Anii 2=Berba 3=Bariba 4=Dendi 5=Fulani, Fulbés, Peuls 6=Lokpa 7=Yom 8=Ditamari 9=Mokolé 10=Waama 11=Foodo 12=Nago 13=Fon 14=Mahi 15=Idaatcha 16=Yoruba 17=Boko 18=Gourmantché 19=Kotocoli 20=Tchabè 21=Autres (préciser :…………….)	
Other sociolinguistic group		

3. **Identification and description of shea local morphotypes**

	Local morphotypes													
	**1**	**2**	**3**	**4**	**5**	**6**	**7**	**9**	**10**	**11**	**12**	**13**	**14**	**15**
**Local name of shea morphotype**														
**Local name traduction**														
**Criteria linked to the name**														

**Trait linked to the name**															
**Characteristics**	**Modality**														
**Fruit yield**	1= Low, 2= Medium, 3= High														
**Butter yield after processing**	1= Low, 2= Medium, 3= High														
**Distribution frequency (abundance)**	1=Widespread<br>2=Rare<br>3=Few<br>4=Unknown														
